# A Survey on Oocyte Donation: Turkish Fertile and Infertile Women’s Opinions

**Published:** 2014-11-01

**Authors:** Aygul Akyuz, Nese Sever, Emre Karasahin, Gulten Guvenc, Suzan Cek

**Affiliations:** 1 School of Nursing, Koc University, Istanbul, Turkey; 2 Department of Obstetrics and Gynecology, Gulhane Military Medical Academy, Ankara, Turkey; 3 School of Nursing, Gulhane Military Medical Academy, Ankara, Turkey

**Keywords:** Third-Party, Assisted Reproduction, Infertility, Oocyte Donation

## Abstract

**Background:**

There are various treatment options for infertility, and new techniques are also being developed as it is an important healthcare problem affecting approximately 15-20% of married couples. The purpose of this study was to evaluate the level of infor- mation of fertile and infertile Turkish women on oocyte donation in order to understand their awareness of the legal, ethical, social and religious issues regarding this technique and to compare these two groups in terms of these variables.

**Materials and Methods:**

This cross-sectional study included infertile women being treated at the assisted reproductive technologies (ART) program of a university hos- pital and women who had presented at the gynecology outpatients department of the same university for routine check-ups and who had no previous history of infertility. After consulting with specialists in the field and searching the related literature, a data collection form having 22 questions for infertile women and 18 questions for fertile women was prepared.

**Results:**

The women were asked whether they would use the oocytes of another woman if necessary. The results showed that 67.6% of the fertile women said they would never want to use this method, while 63.9% of the infertile women stated they may accept to use this method under certain conditions (two distinct answers appeared in the answers, some women stated they would prefer donated oocytes from close relatives, while others stated they would prefer oocytes from total strangers), such as from a close relative or from someone they do not know at all.

**Conclusion:**

Infertile women mentioned that they could use illegal routes if necessary to have a child at much higher rates than stated by fertile women. This shows that desire to have a child is a strong source of motivation in Turkey.

## Introduction

Childbearing is an important goal for marriage and seen as a vital means of stability and satisfaction in married life in many Islamic societies. Thus, being unable to fulfill this pri¬mary goal, an infertile couple is highly likely to be regarded as a failure (1-3). Within this context, infertility is considered as an important public problem which may affect the spouses’ relationships or even threaten their marriage. In addition, the social environment aggravates the situation even further by bringing the couple face to face with society’s expectations (1).

Currently, there are various treatment options for infertility, and new techniques are also being developed as it is an important healthcare problem affecting approximately 15-20% of married couples (4-9). There are a number of different assisted reproduction modalities, some of which involve a third party, such as gamete or embryo donation or surrogate motherhood. Women with ovarian failure were considered irreversibly sterile until approximately 20 years ago, but advances in assisted reproductive technologies (ART) have changed that view. Oocyte donation today offers women with premature ovarian failure or a rapidly diminished ovarian reserve (DOR), a very realistic chance of pregnancy. Current oocyte donation is commonly achieved by *in vitro* fertilization (IVF) using the oocytes retrieved from healthy young donors after controlled ovarian hyperstimulation and the sperm of the recipient’s partner, with the resulting embryos then transferred to the uterus of the recipient. A successful pregnancy established in a recipient woman using donated oocytes was first reported in 1983. Since then, oocyte donation has become a logical extension of assisted reproduction technology (10, 11).

The practice of oocyte donation involves ethical, social, religious, psychological and medical issues. Scant attention has been given to the medical risks of oocyte donation. The risks of oocyte donation mean that special scrutiny should be paid to the treatment of oocyte donors during the donation process. There are several side effects and long term risks that may be associated with being an oocyte donor, which include pain, infection, bleeding as a result of the oocyte retrieval process, premenstrual syndrome like symptoms, ovarian hyperstimulation syndrome (OHSS), and a controversial risk of ovarian cancer from the medications the donor uses. The risks of oocyte donation necessitate the inspection of the treatment of oocyte donors during the donation process (12, 13).

Treatment by oocyte donation, as one of the most contentious issues surrounding assisted reproduction, elicits active debate within many societies with regard to its moral, ethical and religious aspects (8, 14-19). Oocyte donation could be more complicated in Islamic societies where some may even believe that third party reproduction is not permissible under Islamic rules or forbidden by the legislation law. Many countries have passed necessary legal regulations in accordance with their own values and beliefs and started oocyte donation. The rights of the donor, the recipient couple and the child have been determined by law in the countries where the procedure is permitted (15, 16, 20-25). Islam is the dominant religion in Turkey and almost 99% of the population is Muslim (1). According to the law enforcement act on assisted reproduction treatment, only sperm and oocytes obtained from married couples can be legally used in Turkey. Sperm donation, embryo sharing and surrogate motherhood are all forbidden as well as oocyte donation. There are few studies reflecting the opinion of Turkish people toward oocyte donation (17, 26, 27).

At present, there are approximately 120 fertility clinics operating in Turkey. Some of the fertility clinics are sited in public or university hospitals, but the majority of them are established in private hospitals or operate as independent centers. In March 2010, a new version of the regulations, the "Legislation Concerning Assisted Reproduction Treatment Practices and Centers", was introduced in Turkey. In the revised legislation, a number of new restrictions have been declared including limitations regarding the licensing of private IVF centers, specifications on gamete and embryo storage and restrictions on the number of embryos that can be transferred to a patient [only one for women aged under 35 in their first and second cycle of IVF, and a maximum of two embryos for women in their third or subsequent cycles or over 35 years of age (28)].

The use of donor eggs, sperm, or embryos is more of a social or cultural problem than a medical one (29-30). Legal arrangements regarding oocyte donation are, therefore, subject to the cultural beliefs and opinions of the majority of the community about the procedure, individuals are free to use a legal procedure according to their own values. However, the fact that it is illegal can prevent those who want to use it and can push them towards using illegal routes (17, 26, 31). Law makers and legislative bodies define laws in a way that reflects the opinion of the majority. We believe that the opinions of the infertile couples are the most important in this matter. Infertile couples strive to find solutions for both infertility and associated negative effects, and therefore try many treatment options. At this point, we feel that infertile women’s opinions on oocyte donation must be taken into account.

The purpose of study was to evaluate the level of information of fertile and infertile Turkish women on oocyte donation in order to understand their awareness of the legal, ethical, social and religious issues regarding this technique and to compare these two groups in terms of these variables.

The study intended to provide answers to the following questions:

1. What are the opinions of fertile and infertile women regarding oocyte donation and the legal arrangement in Turkey?
2. Would fertile and infertile women accept being an oocyte donor or recipient?
3. Are fertile and infertile women aware of the legal, ethical, religious and social aspects of oocyte donation?

## Materials and Methods

This cross-sectional study was carried out between October 2008 and January 2009 in Ankara, Turkey. Women applying to Gulhane Military Medical Academy gynecology and infertility clinics who were willing to participate and who met the inclusion criteria were included. The inclusion criteria for fertile women were: having conceived spontaneously and having no other gynecological problems, while for infertile women, being under treatment in ART outpatient clinic and having no other additional gynecological problems.

A data collection form was developed by researchers after evaluation of the relevant literature (5, 26, 31). The validity of the content was examined and approved by experienced infertility professionals (the chief of the outpatient ART clinic, the nurse of the outpatient ART clinic, an academic staff who has worked on psychosocial aspects of infertility, and a researcher who is under infertility treatment herself) to confirm the study’s general appropriateness and applicability. The questionnaire consisted of 22 questions for infertile women and 18 questions for fertile women identifying the women’s socio-demographic characteristics (age, level of education, and occupation), history of infertility, and knowledge and opinions about oocyte donation.

In the questionnaire, women were first asked if they had previously heard about oocyte donation, and those who had heard about it were asked to define the procedure. This was done to verify the actual knowledge level of women about the subject. Later, all were informed about the oocyte donation and women then answered the rest of the questionnaire.

The prepared questionnaire was first administered to 10 fertile and 10 infertile women as a pilot study to ascertain whether the items could be easily understood. Since no problems were detected/ reported by women in the pilot stage, the questionnaire was used as is.

The women were provided information on the study in small groups at the waiting hall and those who consented to participate were taken to another room to fill in the data collection forms with face-to-face interviews. A total of 97 infertile women that attended the ART program of Gulhane Military Medical Academy, *In Vitro* Fertilization Center and 105 fertile women with no previous history of infertility were included within the scope of the study.

### Ethical consideration

A detailed report about the study, including the purpose, possible benefits, methods and data collection means, is presented to the Gulhane Military Medical Academy Ethical Committee. Our study was then approved by this ethical committee. All participants were informed and their oral and written consents were taken. After their consent, all participants were interviewed by the researcher for about 20 minutes each, and filled the data collection forms.

### Statistical analysis

The data were analyzed using the "Statistical Package for Social Sciences" (SPSS) version 15.0 (SPSS Inc., Chicago, IL, USA). Descriptive statistics, such as frequency, percentages, means and standard deviations were used to describe the sample and main variables. The appropriateness of the variables (age, duration of marriage and monthly income) was checked by a single sample Kolmogorov-Smirnov test and found to have normal distribution. Chi square test and indepent-samples t test were used to compare infertile and fertile women. Values of p less than 0.05 were considered statistically significant.

## Results

The socio-demographic characteristics of the women are presented in table 1. In table 2, it can be seen that the subject of oocyte donation had previously been heard by 54.6% of the infertile and 41.9% of the fertile women. However, only 40.2% of the infertile women and 18.1% of the fertile women could correctly define "oocyte donation". There was a statistically significant difference between the two groups (p=0.000, χ2=12.04).

The fact that oocyte donation is illegal in Turkey was known by 70.1% of the infertile women and 50.5% of the fertile women. There was a statistically significant difference between the infertile and fertile women regarding their knowledge on illegality of oocyte donation in Turkey (p=0.003, χ2=8.08, Table 2). Both fertile and infertile women felt that current legal arrangement for oocyte donation in Turkey was appropriate at a rate of 71.4 and 57.7%, respectively.

The percentage of women who did not want oocyte donation to be legal in Turkey under any circumstances were 50.5 and 44.3% for the infertile and fertile women, respectively (Table 2).

The women were asked whether they would use the oocytes of another woman if necessary to have a child. About 67.6% of the fertile women said they would never want to use this method, while 63.9% of the infertile women stated that they may want to use this method under certain conditions (such as from a close relative or from someone they do not know at all). The difference between the fertile and infertile women was significant (p=0.000, χ2=20.10, Table 2).

The women were then asked whether they would donate oocytes for someone else if necessary. About 58.1% of the fertile women said they would never want to make a donation, while 55.7% of the infertile women said they may donate their oocytes under certain conditions. The difference between the fertile and infertile women was significant (p=0.03, χ2=3.82, Table 2).

**Table 1:** The sociodemographic characteristics of the women


	Fertile women N=105 X̅ ± SD		Infertile women N=97 X̅ ± SD		P	t

Women’s age (Y)*	32.41 ± 6.19		30.62 ± 3.88		0.016	2.43
Duration of marriage (Y)*	9.25 ± 6.29		6.44 ± 3.37		0.000	3.91
Monthly income (Turkish Lira)*	2768.47 ± 1141.80		1982.29 ± 687.07		0.000	5.87
	**N**	**%**	**N**	**%**	**P**	**χ^2^**
Educational status**						
Primary education	4	3.8	13	13.4	0.000	19.60
High school	41	39.0	57	58.8		
University- or higher	60	57.1	27	27.8		
Employment status**						
Working	75	71.4	35	36.1	0.000	25.39
Not working	30	28.6	62	63.9		


*; Independ-samples t test and **; Chi Square test were used.

**Table 2:** Fertile and infertile women’s knowledge about oocyte donation and its practicabilty in Turkey


	Fertile women N=105		Infertile women N=97			
	N	%	N	%	P	χ^2^

**Knowledge about oocytedonation**						
Heard	44	41.9	53	54.6	0.047	3.27
Not heard	61	58.1	44	45.4		
**Defining "oocyte donation"**						
Defines correctly	19	18.1	39	40.2	0.000	12.04
Can not define	86	81.9	58	59.8		
**Knowledge on illegality ofoocyte donation in Turkey**						
Thinks oocyte donation is legal	-	-	-	-	0.003	8.08
Thinks oocyte donation is illegal	53	50.5	68	70.1		
No opinion	52	49.5	29	29.9		
**Opinions related to currentrules and legislation thatprohibitoocyte donation**						
Oocyte donation must bekept illegal	75	71.4	56	57.7	0.29	4.15
Rules and legislation on oocytedonation must be revised	30	28.6	41	42.3		
**Considering oocyte donation’slegality in the future**						
Never	53	50.5	43	44.3	0.23	0.76
Yes	52	49.5	54	55.7		
**Acceptance of utilizinganother woman’s oocytesto have a child if necessary**						
Never	71	67.6	35	36.1	0.000	20.10
Under some circumstances(Oocytes of a closerelative or from a personwhom she does not know)	34	32.4	62	63.9		
**Desire to be an oocyte donorfor someone else if necessary**						
Never	61	58.1	43	44.3	0.035	3.82
Under some circumstances (For a close relative or a person whom she does not know)	44	41.9	54	55.7		


Chi Square test was used.

In table 3, it can be observed that, both fertile and infertile women stated that oocyte donation may be accompanied by legal, ethical, social and religious problems. The most common (64.8% of the fertile and 41.2% of the infertile women) concern regarding these problems was the emergence of the donor in the following years for financial or emotional demands from the family or the child. The other concerns were the fear of a consanguineous marriage later in life since the biological mother is not known (51.4% of the fertile and 39.2% of the infertile women), the genetic features of the biological mother remaining unknown for both the couple and the child to be born (43.8% of the fertile and 35.1% of the infertile women), and the possibility of elderly couples having children with this method (33% of the fertile and 12.4% of the infertile women).

The percentage of women who stated they would choose adoption in case they could never have a child at all were 81.0% of the fertile and 60.8% of the infertile women in our study. However, 20.6% of the infertile women indicated that they would choose oocyte donation as a second option, even if they knew that it was illegal. The difference between the fertile and infertile women was significant (p=0.00; χ2=19.26, Table 4).

**Table 3:** Fertile and infertile women’s opinions on the advantages and possible problems that oocyte donation may bring


	Fertile women N=105		Infertile women N=97			
	N	%	N	%	P	χ^2^

**This technique will provide infertile couples tohave child and give them ease against theirphysical and psychological problems**	60	57.1	47	48.5	0.13	1.58
**The emergence of the donor later in life wouldlead to financial or emotional demands fromthe family or the child**	68	64.8	40	41.2	0.001	11.21
**I think that couples using this technique willnot feel themselves as real mothers and fathers**	21	20.0	24	24.7	0.26	0.65
**The genetical features of the child to beborn will not resemble his/her mother’sfeatures, thus this would be a problem**	46	43.8	34	35.1	0.130	0.61
**Elder women should not have child throughthis method**	33	31.4	12	12.4	0.001	10.57
**The children born via this method willnever know their exact genetic origins,hence this would result in consanguineousmarriages in the future**	54	51.4	38	39.2	0.054	3.052
**This practice is not appropriate to myreligious beliefs**	15	14.3	18	18.6	0.26	0.67
**This practice is an absolute contradictionwith the Turkish family structure,hence it should not be implemented**	26	24.8	32	33.0	0.12	1.66


Chi square test was used.

**Table 4:** Comparison of fertile and infertile women’s future plans regarding the use of oocyte donation in case they do not have any other choice


	Fertile women N=105			Infertile women N=97				
	N	%	N	%	P	χ^2^

**Prefer to live without a child**	13	12.4	12	12.4	0.000	19.26
**Adopt a child**	85	81.0	59	60.8		
**Go to a country where occytedonation is legal**	5	4.8	6	6.2		
**Try to utilize oocyte donationeven it is illegal**	2	1.9	20	20.6		


Chi square test was used.

## Discussion

This study evaluated the knowledge level and opinions of both fertile and infertile Turkish women on oocyte donation. There have been published studies investigating public opinions about oocyte donation (17), IVF staff attitudes regarding oocyte donation (32), views of infertile women on surrogacy and oocyte donation (27), and gamete donation (26) in Turkey. Approximately half of the fertile and infertile women in the study had heard of oocyte donation. However, only a very small percentage of the fertile women and approximately half of the infertile women could correctly define oocyte donation. This indicates that infertile women seek various treatment options to eliminate infertility and its effects; therefore, they are more informed about the subject. Similar to our results, Khalili et al. (32) reported that half of the Iranian community knew the meaning of oocyte donation. Isikoglu et al. (17) have reported that 29.74% of women knew about oocyte donation in their similar study from Turkey. The high rate of having heard of oocyte donation in our study may be due to the increased awareness of the community in the three years between the two studies.

Both the fertile and infertile women in our study were aware that oocyte donation is illegal in Turkey. They were all approving the illegality of oocyte donation. However, 6 of every 10 infertile women reported that they could donate their oocytes for another woman under certain conditions (the donor is a close relative, never knowing the donor, etc.), and more than half said they could take oocytes from another woman if necessary. The attitude of community towards oocyte donation in different societies is still a controversial issue (32). Of previous researches in Turkey, one had reported that 23.3% of infertile women have stated they could accept oocytes from another woman, while 33.8% have stated they could donate oocytes (26). Another study on fertile women had reported that 82.76% of women have a positive attitude to oocyte donation (17). A study from Sweden (15) had found that one sixth of women felt they could donate oocytes for a woman they did not know, while another study (33) had reported 66% of the subjects stating they could donate oocytes for their siblings. A study from Iran (32) had reported that there were not so much difference between Christian and Muslim communities towards their reaction to oocyte

donation and the majority of Iranian public supported oocyte donation as an alternative way of overcoming infertility.

These results indicate that the percentage of Turkish infertile women with a positive attitude towards oocyte donation is constantly increasing and the method is today deemed to be more acceptable both in Turkey and in other countries.

Fertile women have a more unfavorable approach both to oocyte donation and acceptance compared to infertile women. Fertile women wanted oocyte donation to be kept illegal in the future and stated they would not use it even if necessary. However, although infertile women wanted it to be kept illegal in the future, they felt they could use it if necessary. This result is important as it indicates that infertile women feel a conflict between oocyte donation and the desire to have a child. Fertile women have a more negative attitude towards oocyte donation probably because they are not faced with infertility (16).

Approximately half of fertile and infertile women stated that oocyte donation would enable infertile couples to have children, and therefore provide physical and psychological comfort for them. Svanberg et al. (15), Purewal and Vanden Akker (34) have also reported that oocyte donation is a useful method for childless couples. However, both the fertile and infertile women in our study felt that legalization of oocyte donation could lead to ethical, legal, social and religious problems. This result could be related to ethical, sociocultural and religious characteristics of the Turkish society.

The percentage of women who believed oocyte donation would harm religious values or the family structure was quite low in our study. Isikoglu et al. (17) have similarly reported that less than half the participants stated their beliefs prevented oocyte donation.

Infertile women in the current study mentioned they could use illegal routes if necessary to have a child at much higher rates than fertile women. This shows that desire to have a child is a strong motivation in Turkey. To conclude, it is demonstrated that there are infertile couples who try to find and willing to use third-party assisted reproduction techniques, although illegal in this country. It is possible (and known) that some couples travel abroad to certain countries where oocyte donation is legal to make use of the method.

However, employing these techniques without vast information could harm both the couple- the family and the child born as a result. The infertility nurse also has a responsibility to inform the infertile couple about all procedures, whether legal or illegal. The nurse needs to know the characteristics of the group, he/she, is communicating with, so that information can be provided properly. These characteristics would encompass the cultural values that could influence the final decision.

This study has been conducted in an infertility outpatient center in the capital city of Turkey, Ankara. Therefore, as a limitation, the results deriven should not be generalized.

The religious beliefs of the subjects could have influenced the answers given to the questionnaire. We would suggest "larger and possibly multi centered researches" on the topic, including subjects from various religious and cultural societies. Since the majority of the population believes Islam in our country, and since other religious societies are rather concentrated in small groups in various cities, it was not possible for us as a small group of researchers to reach a larger pool of data from different religions. Our results, therefore, reflects the opinions of a Turkish population who all believe Islam.

Finally only fertile and infertile women were included in this study. But the treatment process and the choice of therapeutic options necessitates the husbands’ opinion in the decision making. Therefore, it would be interesting to search husbands knowledge and approach to oocyte donation in future studies. Missing the male counterpart’s opinions may, therefore, be considered another limitation of this study.

## Conclusion

This study shows that approximately half of respondents had heard of oocyte donation; however, only a very small percentage of fertile women and approximately half of infertile women could correctly define it. The majority of both the fertile and infertile women were aware that oocyte donation is illegal in Turkey. Infertile women have a more favorable approach and support oocyte donation as an alternative route for childless couples, compared to fertile women. Infertile women also mentioned that they could use illegal routes if necessary to have a child at much higher rates than stated by fertile women. This shows that the desire to have a child is a strong source of motivation in Turkey. Health care professionals need to be aware of the emotional and psychosocial impact of being childless in Turkish society.

The explanation of the current legal status in Turkey and the advantages and disadvantages of donation regarding the couple and the child to be born should be included in this presentation. The healthcare staff should provide the necessary guidance after checking the motivation of the couple.
